# Regulation of Flowering Time by Improving Leaf Health Markers and Expansion by Salicylic Acid Treatment: A New Approach to Induce Flowering in *Malus domestica*

**DOI:** 10.3389/fpls.2021.655974

**Published:** 2021-07-19

**Authors:** Kamran Shah, Na An, Svetlana Kamanova, Lijuan Chen, Peng Jia, Chenguang Zhang, Muhammad Mobeen Tahir, Mingyu Han, Yuduan Ding, Xiaolin Ren, Libo Xing

**Affiliations:** ^1^College of Horticulture, Northwest A&F University, Yangling, Shaanxi, China; ^2^College of Food Science and Engineering, Northwest A&F University, Yangling, Shaanxi, China

**Keywords:** apple, antioxidant, chlorophyll, flowering, leaf pigments, mediator, porphyrin

## Abstract

In the external coincidence model, internal and external molecular signals, provided by the circadian clock and sunlight, respectively, are required to induce flowering. Salicylic acid (SA) applications during floral induction have multiple effects. In the current study, *Malus* × *domestica* plants were exposed to SA during the flower-induction stage to analyze the effect on various health markers and flowering. A total of 56 equal-sized Fuji/M9 trees that were about 7 years old were randomly divided into two groups. The first group (SA-treated) was sprayed with 4 mM SA solution, while the second group was sprayed with distilled water which served as control (CK). The SA applications increased various leaf pigments. Abiotic stress markers were increased in CK during the flower-induction stage. In the SA-treated group, non-enzymatic antioxidants increased, whereas in the control group, enzymatic antioxidants increased during the flower-induction stage. Histo-morphometric properties of leaves were significantly improved in the SA-treated group. The relative expression of the mRNA levels of *MdMED80*, *−81*, *−3*, and *−41* were significantly increased in SA-treated leaves, leading to an early and increased flowering phenotype. Thus, SA increased leaf expansion and health-related marker levels, which lead to early induction of flowering in *M. domestica*. Overall, our work established a role for leaf health assessments in the regulation of flowering in *M. domestica*.

## Introduction

Salicylic acid (SA) is a natural phenolic plant hormone that is a leading candidate for improving salinity tolerance (Nazar et al., 2015). It plays vital physiological roles in nutrient uptake, growth, development, thermogenesis, ion absorption, flower induction, transport, stomatal movement, photosynthesis, and transpiration, which affect plant performance (Vlot et al., 2009; Hayat et al., 2010). Moreover, SA applications induce antioxidative enzyme activity, which in turn increases plant resistance to NaCl-related toxicity (He and Zhu, 2008). Exogenous SA sprays improve the morphometric properties of some vegetables, such as cucumber (Yildirim et al., 2008), tomato (Stevens et al., 2006), and summer squash (Elwan and El-Shatoury, 2014). Salicylic acid is also involved in endogenous signal transduction and mediates defense system responses against pathogens (Hayat and Ahmad, 2007) by upregulating disease-related proteins (Van Huijsduijnen et al., 1986). Therefore, it is important to understand the roles of exogenous SA applications during the flower-induction stage (FIS) in *Malus* × *domestica*.

*Malus domestica* is a fruit tree that is grown commercially in temperate regions worldwide (Velasco et al., 2010). However, alternate bearing in “Fuji”/”M9” trees results in the buds failing to regulate flowering during the FIS. Many approaches have been used to generate genetic, hormonal, physiological, and morphological indices of buds during the FIS. During the FIS, the initial flowering signal initiates at the leaf vasculature and then passes through the phloem to the buds (Corbesier et al., 2007). Leaf health-related markers represent salient traits that regulate whole-plant vigor and produce the primary signal for flower initiation. Healthy leaves readily detect any changes in photoperiod or exogenous/endogenous stimuli and transmit signals to the shoot apical meristem (SAM) (An et al., 2004; Corbesier and Coupland, 2006; Turck et al., 2008). The SAM is configured by changes in cell division, resulting in the floral primordia forming flowers rather than leaves. When *Arabidopsis* is exposed to long-day conditions it activates flowering, confirming that signals from leaves initiate flowering (Fornara et al., 2010). In maize, the presence of four to six leaves is required for the meristem to produce flowers, whereas in impatiens, the continued assembly of an inductive signal from leaves is required (Irish and Nelson, 1991; Irish and Jegla, 1997; Pouteau et al., 1997). It is important to understand the roles of exogenous SA applications during the FIS in *M. domestica* leaves. In addition, the primary tissues in which flowering-time genes are required to activate flowering have not been extensively studied in *M. domestica*. However, genes that initiate in leaves, and those that initiate in the meristem of pea and maize, have been distinguished (Colasanti and Sundaresan, 1997; Weller et al., 1997). Thus, we hypothesized that leaf health is a prominent factor in photoperiodic flowering. Healthier leaves capture more light to activate the primary gene signal in the leaf vasculature, and the signal passes through the phloem to the SAM to induce flowering (Corbesier et al., 2007).

Leaves are the most important and exposed parts of plants, and they support the plant’s growth and developmental functions. Leaves also sense a variety of environmental stimuli, such as light and temperature, to initiate flowering. Mediator proteins are found in all eukaryotes (Boube et al., 2002; Kornberg, 2005; Bäckström et al., 2007), being required for the transcriptional regulation of RNA polymerase II (Blazek et al., 2005). Previously, mediator proteins were biochemically purified from *Arabidopsis* (Bäckström et al., 2007) and rice (Bourbon, 2008; Mathur et al., 2011). The mediators phytochrome and flowering time 1 (*PFT1*) and cryptic precocious (*CRP*) have been reported as novel flower regulatory genes in *Arabidopsis* (Imura et al., 2012; Iñigo et al., 2012). Phytochrome proteins found in leaves encode light-absorbing pigments that control photo morphogenetic features in plants, such as stem elongation, seed germination, pigment formation, leaf expansion, chloroplast development, and flowering. Among these photoreceptors, *PFT1* is a key component of the light-quality pathway, and it works downstream of phytochrome B (PhyB) to adjust the expression of flowering locus T (FT), which regulates flowering time in plants (Cerdán and Chory, 2003). In response to light, the photoperiodic pathway genes gigantea (*GI*) and constans (*CO*) induce flowering (Kim et al., 2008; Wollenberg et al., 2008), whereas *PFT1* promotes flowering through CO-dependent and -independent mechanisms (Iñigo et al., 2012). Additionally, *CRP* is a newly discovered mediator gene for flowering that works with FT both downstream and upstream of the key flowering genes (Imura et al., 2012) in the leaves (Takada and Goto, 2003; Kobayashi and Weigel, 2007; Adrian et al., 2010; Imaizumi, 2010). The FT protein is expressed in the leaf, moves through the phloem to the SAM, and then, it triggers several positive floral regulators to set flower formation (Corbesier et al., 2007; Jaeger and Wigge, 2007; Mathieu et al., 2007; Notaguchi et al., 2008). *CRP* is required for regulation of fruitfull, suppressor of overexpression of constans 1, apetala1, twin sister of FT, FT, and bZIP protein FD, as well as the downregulation of flowering locus C (Imura et al., 2012).

Accurate daylight signals upregulate the FT protein, which triggers flowering (Xing et al., 2016, 2019). Three factors contribute to a high FT protein production. First, the coordinated expression of flavin-binding (FKF1), and GI by the circadian clock. Second, the stabilization of the FKF1–GI complex by light, and finally, the stabilization of the CO protein by light (Song et al., 2014). However, this whole process takes place in the leaves. Once FT is produced, it moves toward the buds and triggers the developmental processes that lead to flowering induction (Nelson et al., 2000; Sawa et al., 2007; Song et al., 2014). Thus, the leaf maintains the most important key signal that instructs buds when and how much to flower. However, leaf morphometric health assessments in response to SA applications during the FIS have been ignored in *M. domestica*. We hypothesized that SA treatments positively affect the leaf morphometric and health-related markers, thereby improving flowering induction. Therefore, this study was designed to investigate the roles of SA’s effects on morphometric and health-related markers of *M. domestica* leaf in regulating flowering time during the FIS.

## Materials and Methods

### Experimental Site and Climatic Condition

The experimental site is located at Haisheng Modern Agriculture Company, Limited (Qianyang County, Baoji, Shaanxi, 34° 64′ N, 107° 13′ E) (Fan et al., 2019). Qianyang in the Shaanxi region of China has the best apple,-growing areas, where climatic conditions are favorable for apple production. In Qianyang, summers are humid, warm, and partly cloudy. Winters are dry, very cold, and mostly clear. Around the year, the temperature typically varies from −6.11°C to 29.44°C and is rarely below −10.55°C or above 33.88°C. Detailed monthly meteorological data of the experimental site is represented in Supplementary Table 2.

### Experimental Design, Plant Treatment, and Sampling

A total of 56 equal-sized Fuji/M9 trees that were about 7 years old were selected in the apple farm. The trees were randomly and equally divided into two groups. We collected the fresh leaf samples adjacent to the buds at each time point of days after full bloom (DAF). We calculated 4-mM salicylic acid for 10 l of distilled water by using the 2021 GraphPad QuickCalcs tool^1^. The samples from day 0 from both groups were collected, and then the first group (SA treated) was sprayed with 4 mM SA solution, while the second group was sprayed with distilled water which served as control (CK). Both groups were sprayed two times on April 5, 2018, and 2 days later on April 7, 2018. Fresh leaves were collected eight times from each group at 10 days interval starting from 0 to 70 DAF. Half of the freshly collected leaves were directly frozen in liquid nitrogen and stored at −80°C. The remaining leaves were stored at 4°C for morphometric studies.

### Morphometric Studies

Fresh leaf samples were harvested and sent to the laboratory at College of Horticulture, Northwest A&F University, Yangling, China for morphometric analysis. A total of 10 leaves from each group at each DAF were scanned using an Epson Perfect scanner (Model: V330 Photo, Epson, Indonesia) and then Leaf Auto-Compute software was used to calculate leaf width (cm), length (cm) and leaf area (cm^2^). An Ohaus digital scale (OHAUS Scale Corporation, Florham Park, NJ, United States) was used to observe leaf weight. In addition, leaves were placed in an oven at 60°C for 24 h to measure dry matter content (DMC).

### Stress Marker Analysis

Freeze dried leaf samples were crushed into fine powder and stress marker were analyzed in the samples (Velikova et al., 2000).

### Determination of Hydrogen Peroxide (H_2_O_2_) Content

Hydrogen Peroxide content was measured according to the previous protocol (Sergiev et al., 1997; Shi et al., 2005). A total of 0.5 g leaf tissue was homogenized and the absorbance of the supernatant at 390 nm was determined by using a spectrophotometer (Model: UV-1201, Shimadzu Spectrophotometer, Japan). The standard y-curve was used to calculate the H_2_O_2_ concentration.

### Determination of Malondialdehyde (MDA) Content

MDA was assessed to determine lipid peroxidation by thiobarbituric acid in leaves tissue (Velikova et al., 2000).

### Enzymatic Antioxidant

To measure enzymatic antioxidants, 0.5 g of leaves were crushed, homogenized with 5 mL of potassium phosphate buffer (10 mM, pH 7.0) and polyvinylpyrrolidone (4% w/v), and centrifuged at a rate of 12,000 × *g*, 4°C for 30 min (Sorvall ST16R, Thermo, United States). The upper phase was used to determine CAT (Gong et al., 2001), POD (Fernández-Trujillo et al., 2003) and SOD (Agarwal and Shaheen, 2007). POD activity was calculated by spectrophotometer and the increased absorbance at 470 nm was detected in phosphate buffer containing guaiacol (1 mM) and H_2_O_2_ (0.5 mM). One unit of POD is the amount of enzyme that increases the absorbance by 0.01/min. CAT activity was measured by observing a decrease in absorbance of phosphate buffer (50 mM, pH 7.5) with H_2_O_2_ (20 mM) at 240 nm. One unit of CAT is the amount of enzyme used at 1 mM H_2_O_2_ per min. The unit of SOD is the amount that reduces the absorbance value to 50% compared to the control (without enzyme).

### Non-enzymatic Antioxidants

The concentration of ascorbic acid in leaves was measured according to the previous protocol (Tausz et al., 2004). Briefly, leaf tissue was homogenized with 1.5% (w/v) metaphosphoric acid containing 1 mM ethylenediaminetetraacetic acid, and the extract was subjected to HPLC analysis using water/methanol (3/1, v/v) with 0.05% (w/v) sodium dihydrogen phosphate monohydrate (pH 3.6), 1 mM hexadecylammonium bromide at a flow rate of 1 mL/min for 20 min and photodetection at 248 nm.

### Leaf Pigment Analysis

For leaf pigments analysis, we used an acetone reagent to extract the leaf pigments and perform the extraction under subdued light as described in our previous report (Shah et al., 2017, 2018, 2019a,b, 2020).

### Histology and Microscopy

Histological analysis of leaves was performed as described in our previously published reports (Shah et al., 2019a, 2020).

### RNA Extraction, cDNA Synthesis, and mRNA Expression Analysis by RT-qPCR

Next we extracted total RNA from leaf samples by Plant Total RNA Isolation Kit Plus (Foregene, Chengdu, China) following the manufacturer’s instructions. RNA concentration was determined using a Nano-drop (1000 spectrophotometer NanoDrop Technologies, Wilmington, DE, United States). First strand cDNA was produced using 1 μg total RNA by using a PrimeScript RT Reagent Kit with gDNA Eraser (Takara Bio, Shiga, Japan). cDNA concentration was determined and diluted to 200 μg. *PFT1* (*MdMED80* and *MdMED81*) and *CRP* (*MdMED3* and *MdMED41*) are highly involved in flowering (Imura et al., 2012; Iñigo et al., 2012), according to our phylogenetic analysis (unpublished) (Supplementary Figure 2), *MdMED80*, *MdMED81, MdMED3*, and *MdMED41* were clustered within same phylogeny clade, possessing close homology with *MdMED2*, *MdMED7*, and *MdMED72*, and these genes were selected for RT-qPCR analysis. We designed primers using Premier 6.0 Biosoft International (Supplementary Table 1) and protein modeling and prediction analysis were constructed (Supplementary Figure 3) using Phyre2 web portal http://www.sbg.bio.ic.ac.uk/phyre2/html/page.cgi?id=index (Kelley et al., 2015). The RT-qPCR reactions were prepared with SYBR Green qPCR Kit (TaKaRa) and Bio-Rad CFX 134 Connect Real-Time PCR Detection System (Bio-Rad, Hercules, CA, United States) with cycling protocol were as 95°C for 3 min, 40 cycles at 95°C for 15 s, 58°C for 20 s, and 72°C for 20 s. For normalization, the apple ACTIN gene was used (Wang et al., 2020; Tahir et al., 2021). Three biological replicates were performed for each sample with three technical replicates and the 2^–Δ^ ^Δ^ ^Ct^ method was used to calculate relative gene expression (Livak and Schmittgen, 2001).

### Statistical Analysis

We use GraphPad PRISM version 7.00 for windows GraphPad Software, San Diego, CA, United States, www.graphpad.com to statistically analyze our data. Student *t*-test was used to analyze the data. Results were expressed as means ± SD, significance values were presented as: ^∗^*p* < 0.05; ^∗∗^*p* < 0.01; ^∗∗∗^*p* < 0.001; ^****^p < 0.0001; while non-significant (ns) (*p* > 0.05).

## Results

### Leaf Morphology

**FIGURE 1:**
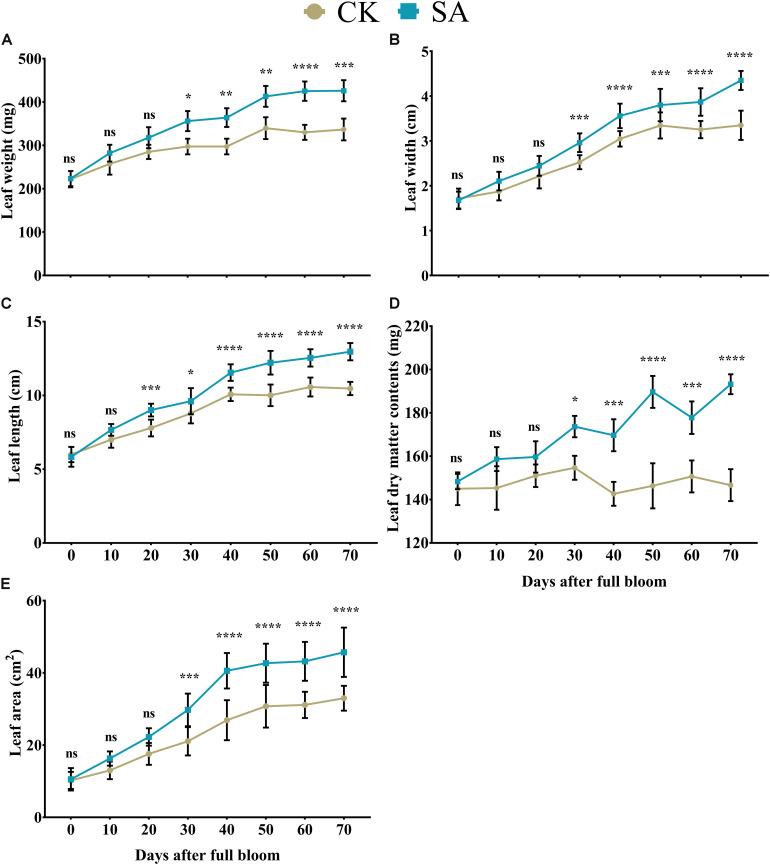
Effects of salicylic acid treatments on *Malus* × *domestica* leaf morphology during the flower-induction stage. **(A)** Leaf weight (mg), **(B)** Leaf width (cm), **(C)** Leaf length (cm), **(D)** Leaf DMC (mg), and **(E)** Leaf area (cm^2^). Results were expressed as means ± SD (*n* = 10), significance values were presented as: *p < 0.05; **p < 0.01; ***p < 0.001; ****p < 0.0001; while non-significant (ns) (*p* > 0.05).

We primarily investigated the morphometric aspects of the leaf in response to SA treatment. At 0–20 DAF, we observed no significant difference in leaf weight (Figure 1A), width (Figure 1B), dry matter content (Figure 1D), and area (Figure 1E) between CK and SA treated groups, while these parameters were significantly increased at 30–70 DAF in SA treated group. Leaf length was not influenced by SA at 0–10 DAF, while at 20–70 DAF the leaf length was significantly increased in SA treated group (Figure 1C). These results suggested that SA has enhanced leaf growth during FIS. Since the leaf in CK treated groups showed reduced growth, we next examined the abiotic stress marker such as H_2_O_2_ and MDA.

### Abiotic Stress Markers

**FIGURE 2:**
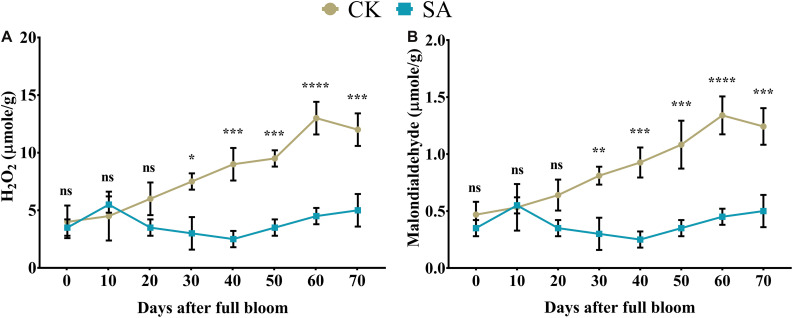
Quantification of hydrogen peroxide and malondialdehyde in salicylic acid-treated and control *Malus* × *domestica* leaves during the flower-induction stage. **(A)** H_2_O_2_ (μmole/g) and **(B)** Malondialdehyde (μmole/g). Results were expressed as means ± SD (*n* = 3), significance values were presented as: *p < 0.05; **p < 0.01; ***p < 0.001; ****p < 0.0001; while non-significant (ns) (*p* > 0.05).

Next, we sought to determine H_2_O_2_ and MDA contents in leaves of CK and SA treated plants. We found no significant difference in H_2_O_2_ (Figure 2A) and MDA content (Figure 2B) of CK and SA treated groups at 0–20 days of DAF, while at 30–70 DAF, H_2_O_2_, and MDA were significantly increased in the CK group. This indicated that the leaves of CK plants were experiencing some environmental stress such as heat and ultraviolet radiation (Supplementary Table 2) during the FIS. To confirm this phenomenon, we next sought to analyze antioxidant activity in leaves which might be affected in response to the increased ROS activity in CK treated groups.

### Enzymatic Antioxidant

**FIGURE 3:**
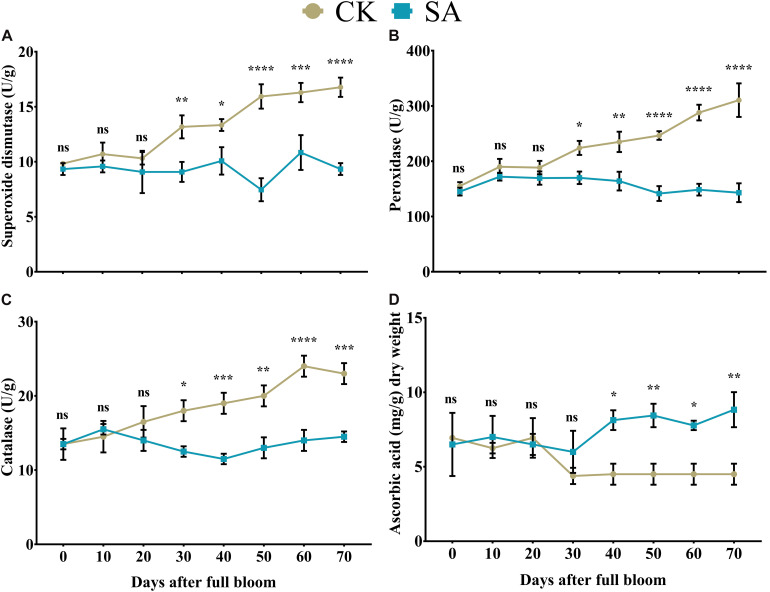
Quantification of enzymatic antioxidants and non-enzymatic antioxidants in salicylic acid treated and control *Malus* × *domestica* leaves during the flower-induction stage. **(A)** Superoxide dismutase (U/g), **(B)** Peroxidase (U/g), **(C)** Catalase (U/g), **(D)** Ascorbic acid (mg/g) dry weight. Results were expressed as means ± SD (*n* = 3), significance values were presented as: *p < 0.05; **p < 0.01; ***p < 0.001; ****p < 0.0001; while non-significant (ns) (*p* > 0.05).

The release of enzymatic antioxidants is a dynamic process in plants that can prevent damage associated with various stressors (Gill and Tuteja, 2010). Initially, we found no significant difference in the SOD- (Figure 3A), POD- (Figure 3B), and CAT-content (Figure 3C) between CK and SA treated groups at 0–20 DAF. However, at 30–70 DAF, SOD- (Figure 3A), POD- (Figure 3B), and CAT-contents (Figure 3C) were significantly upregulated in CK treated plants. This showed that the control group *M. domestica* leaves in native field conditions were in stress during the FIS.

### Non-enzymatic Antioxidant

Stability between ROS and non-enzymatic antioxidants is essential for maintaining plant health. Ascorbic acid take a significant part in plant leaf adaptation to a variety of physiological responses by regulating a cascade of spontaneous oxidation (Khan et al., 2011). Initially at 0–30, no significant difference was observed between CK and SA treated plants, whereas in SA treated group it was significantly upregulated at 40–70 DAF (Figure 3D).

### Leaf Physiological Parameters

After observing the morphological influences of a leaf treated with SA, we sought to detect the leaf physiological parameters such as leaf chlorophyll pigments and their derivatives.

### Chlorophyll Content

**FIGURE 4:**
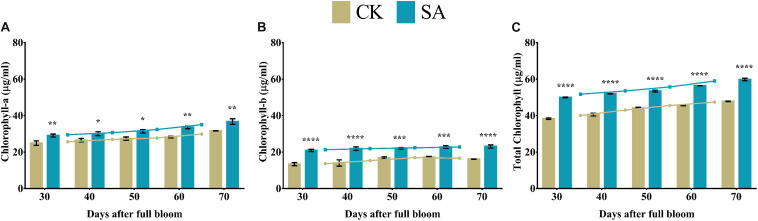
Chlorophyll contents of *Malus* × *domestica* leaves in salicylic acid treated and control group during flower-induction stage. **(A)** Chlorophyll-a (μg/ml), **(B)** Chlorophyll-b (μg/ml), and **(C)** Total chlorophyll (μg/ml). Results were expressed as means ± SD (*n* = 3), significance values were presented as: *p < 0.05; **p < 0.01; ***p < 0.001; ****p < 0.0001; while non-significant (ns) (*p* > 0.05).

Weak chloroplast pigments are the primary consequence of plants indicating stunted leaf health and growth. Next, we examined various leaf pigments in the leaves of CK and SA treated plants at 30–70 DAF. We found that SA treatment upregulated the level of different leave pigments. Chlorophyll-a (Figure 4A), chlorophyll-b (Figure 4B), and total chlorophyll (Figure 4C) were significantly upregulated in SA treated plants from 30 to 70 DAF.

### Porphyrin Content

**FIGURE 5:**
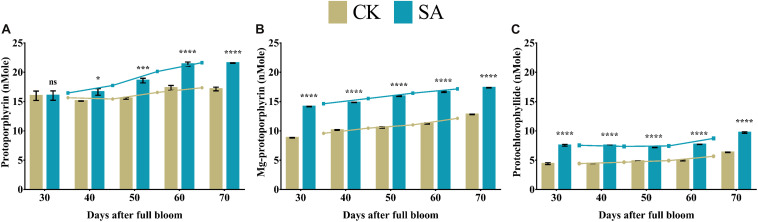
Porphyrin contents of *Malus* × *domestica* leaves in salicylic acid treated and control group during flower-induction stage. **(A)** Protoporphyrin (nMole), **(B)** Magnesium protoporphyrin (nMole), **(C)** Protochlorophyllide (nMole). Results were expressed as means ± SD (*n* = 3), significance values were presented as: *p < 0.05; ***p < 0.001; ****p < 0.0001; while non-significant (ns) (*p* > 0.05).

Protoporphyrin, magnesium protoporphyrin, and protochlorophyllide are collectively called porphyrin, are critically important for chlorophyll biosynthesis. Protoporphyrin- (Figure 5A), magnesium protoporphyrin- (Figure 5B), and protochlorophyllide-contents (Figure 5C) were significantly upregulated in SA treated plants compared to CK from 30 to 70 DAF, except protoporphyrin at 30 DAF, which was not significant.

### Chlorophyllide Contents

**FIGURE 6:**
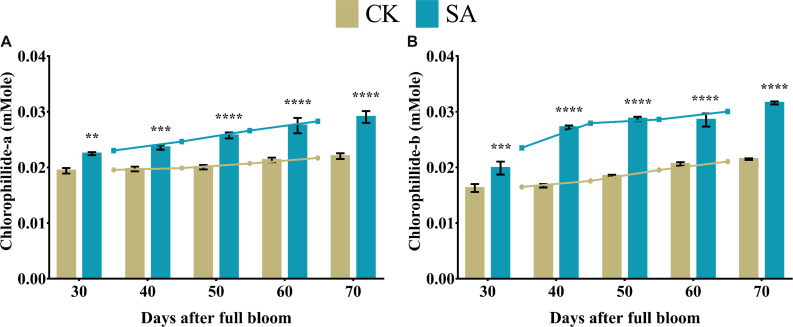
Chlorophyllide content of *Malus* × *domestica* leaves in salicylic acid treated and control group during flower-induction stage. **(A)** Chlorophyllide-a (mMole) and **(B)** Chlorophyllide-b (mMole). Results were expressed as means ± SD (*n* = 3), significance values were presented as: **p < 0.01; ***p < 0.001; ****p < 0.0001; while non-significant (ns) (*p* > 0.05).

Chlorophyllide-a and chlorophyllide-b are biosynthetic precursors of chlorophyll-a and chlorophyll-b, respectively. Therefore, the main interest of these compounds lies in the biosynthesis of chlorophyll in plants. Consequently, we analyzed the level of chlorophyllide contents in leave of *M. domestica* treated with SA and CK. Chlorophyllide-a (Figure 6A) and chlorophyllide-b (Figure 6B) were found significantly upregulated in SA treated plants at 30–70 DAF.

### Pheophytin Content

**FIGURE 7:**
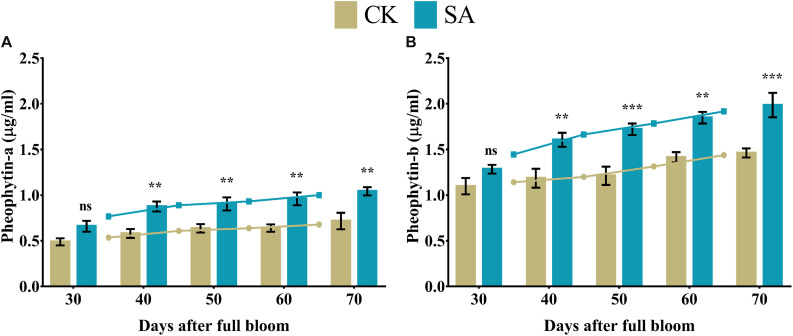
Pheophytin contents of *Malus* × *domestica* leaves in salicylic acid treated and control group during flower-induction stage. **(A)** Pheophytin-a (μg/ml), **(B)** Pheophytin-b (μg/ml). Results were expressed as means ± SD (*n* = 3), significance values were presented as: **p < 0.01; ***p < 0.001; while non-significant (ns) (*p* > 0.05).

Pheophytins are formed by weak acidification from chlorophyll, which lacks Mg^2+^ at the center. We observed a significant upregulation of pheophytin-a (Figure 7A) and pheophytin-b levels (Figure 7B) in SA treated plants at 40–70 DAF compared to CK.

### Carotenoid Contents

**FIGURE 8:**
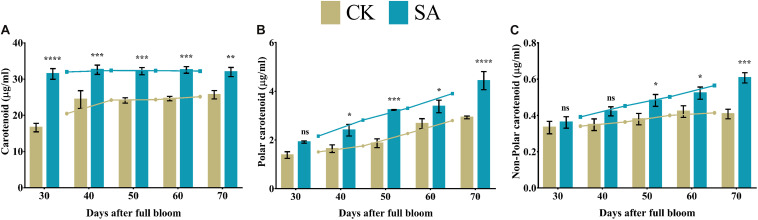
Carotenoid contents of *Malus* × *domestica* leaves in salicylic acid treated and control group during flower-induction stage. **(A)** Carotenoid (μg/ml), **(B)** Polar carotenoid (μg/ml), **(C)** Non-polar carotenoid (μg/ml). Results were expressed as means ± SD (*n* = 3), significance values were presented as: *p < 0.05; **p < 0.01; ***p < 0.001; ****p < 0.0001; while non-significant (ns) (*p* > 0.05).

We found significantly higher levels of carotenoids in SA treated plants from 30 to 70 DAF compared to CK (Figure 8A). While no-significant difference was found in polar carotenoid at 30 DAF and non-polar carotenoid at 30–40 DAF. The rest polar carotenoid and non-polar carotenoid were significantly upregulated in SA treated plants (Figures 8B,C).

### Leaf Histology

**FIGURE 9:**
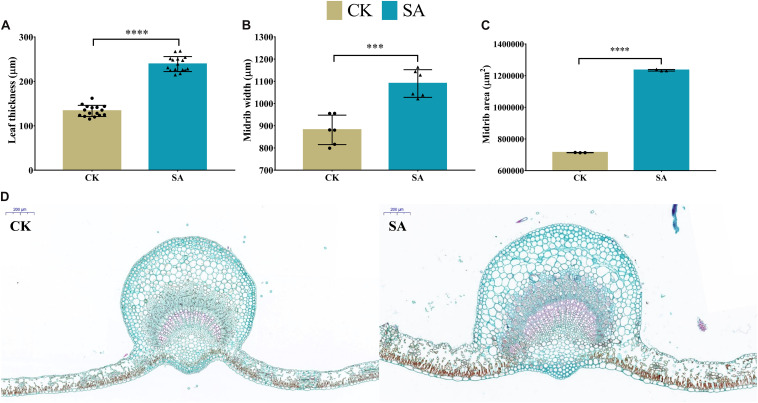
Leaf histological comparison between salicylic acid-treated and control *Malus* × *domestica* leaves at 70 days after full bloom during the flower-induction stage. **(A)** Leaf thickness (μm) (*n* = 16), **(B)** Midrib width (μm) (*n* = 6), **(C)** Midrib area (μm^2^) (*n* = 3), and **(D)** Anatomical histology of transverse section of the CK (left) and SA treated (right) leaf at 70 DAF with scale 200 μm at top left corner. Results were expressed as means ± SD, significance values were presented as: ^∗∗∗^*p* < 0.001; ^****^p < 0.0001; while non-significant (ns) (*p* > 0.05).

To elucidate the response of SA to cell-based phenotypic traits during the flower-induction phase, histological analysis of leaves was performed to observe the micro phenotypes at the cellular level. The CK and SA treated laves cross sections of leaf blade are shown in Figure 9D. We observed the upregulation of leaf thickness 238.9 μm (Figure 9A), midrib width 1,090 μm (Figure 9B), and midrib area 1,234,351 μm^2^ (Figure 9C) in SA treated plants, while the minimums of 133.4 μm, 881.3 μm, and 713,890 μm^2^ were observed in CK treated plants, respectively. Figure 9D represents the anatomical images of CK and SA treated leaves. We used ImageJ software to measure various variables such as leaf thickness, midrib width, and midrib area.

### Gene Expression Pattern

**FIGURE 10:**
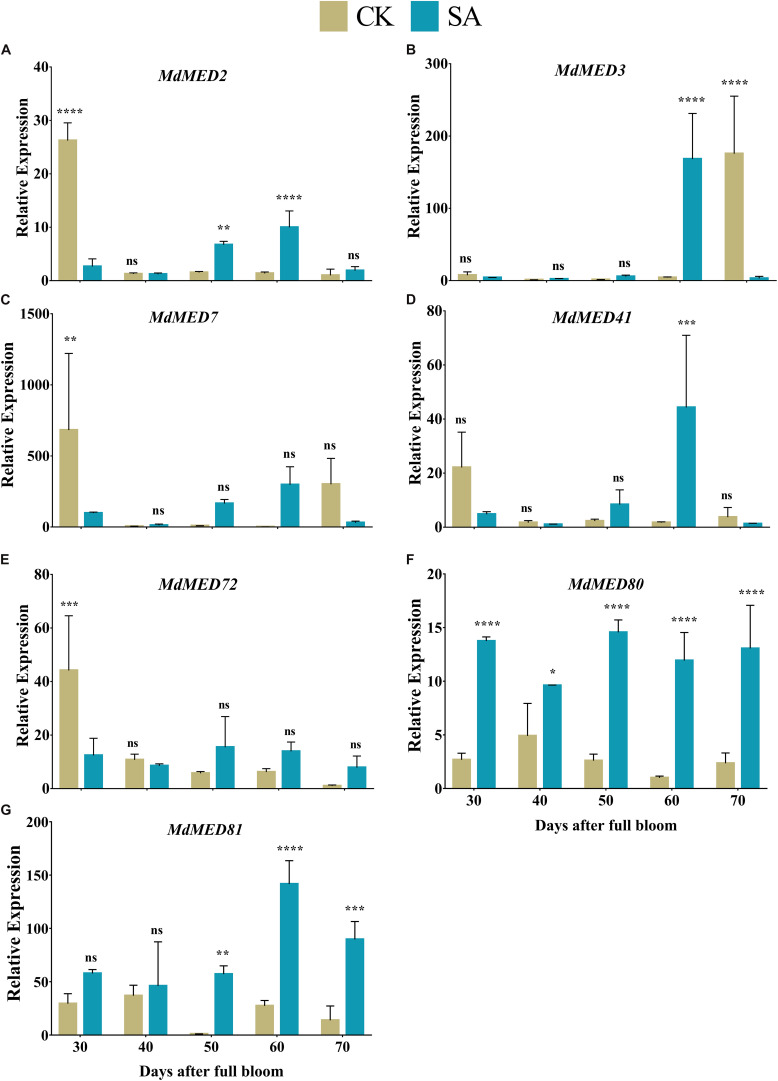
Quantification of gene expression levels in salicylic acid treated and control *Malus* × *domestica* leaves during the flower-induction stage. **(A)** *MdMED2*, **(B)** *MdMED3*, **(C)** *MdMED7*, **(D)** *MdMED41*, **(E)** *MdMED72*, **(F)** *MdMED80*, and **(G)** *MdMED81*. Results were expressed as means ± SD (*n* = 3), significance values were presented as: *p < 0.05; **p < 0.01; ***p < 0.001; ****p < 0.0001; while non-significant (ns) (*p* > 0.05).

*MdMED80* and *MdMED81* are known to be involved in the regulation of flowering time in response to light quality (Aukerman et al., 1997; Devlin et al., 1998; Cerdán and Chory, 2003). To elucidate the response of leaf expansion carried by SA, we performed the RT-qPCR of potential homologs of *PFT1* and *CRP* genes. The relative expression pattern revealed the confirmatory evidences that support our hypothesis regarding floral induction. *MdMED2* (Figure 10A) was found upregulated in CK treated plants at 30 DAF, however, it was significantly increased in SA treated plants at 40–50 DAF. *MdMED7* (Figure 10C) and *MdMED72* (Figure 10E) was significantly upregulated at 30 DAF in CK treated plants and became non-significant at 40–70 DAF. The *MdMED80* (Figure 10F), and *MdMED81* (Figure 10G) were significantly upregulated in SA treated plants at 30–70 and 50–70 DAF, respectively. *MdMED3* (Figure 10B) and *MdMED41* (Figure 10D) homologs of *CRP* was significantly increased at 60 DAF in SA treated plants. This demonstrated that under photoperiodic flowering, leaf health and expansion is key factor that contributes to absorption of accurate ratio of red to far-red light signal and activate flowering.

## Discussion

The growth and expansion of leaves enables plants to capture the appropriate ratio of red to far-red light signals and triggers a variety of processes, such as plant growth, photosynthetic mechanisms, and flowering. Leaf vigor and growth involve responses of many cell types to various environmental and internal cues (Shah et al., 2020). Here, SA-treated plants showed greater dry matter contents, which ultimately triggered leaf growth and expansion during the FIS (Figure 1). Salicylic acid treatments promote leaf growth in barley (Pancheva et al., 1996), leaf weight, leaf number (Hayat et al., 2005), leaf area and DMC in wheat (Hussein et al., 2007), as well as leaf pigments and the photosynthetic rate in maize (Khodary, 2004). Moreover, the leaf growth-promoting properties of SA are associated with changes in hormonal level in rosemary (Najafian et al., 2009), wheat (Shakirova et al., 2003), soybean (Gutiérrez-Coronado et al., 1998), and maize (Gunes et al., 2007).

Pattern recognition receptors identify conserved motifs in pathogens and represent systemic acquired resistance, which triggers an immune response through the activation of pathogenesis-related genes that require an SA accumulation (Conrath, 2006). This supports our findings regarding abiotic stress markers (Figure 2) and enzymatic antioxidative activities (Figure 3) in SA-treated plants that regulate leaf growth and increase the leaf area (Van Huijsduijnen et al., 1986; Hayat and Ahmad, 2007). Enhanced leaf growth, along with increased carotenoid and anthocyanin contents, result from the parallel increase in total leaf antioxidative activity (Eraslan et al., 2007).

Owing to the slow growth, small size and delicacy of CK-treated leaves during the FIS, stress-related marker increased (Figure 2), which increased the leaf temperature and caused heat stress. However, SA functions as a plant growth regulator and alleviates temperature under heat-stress conditions by influencing various physiological processes and biochemical reactions (Raskin, 1992; Wang et al., 2010; Nazar et al., 2011). During the FIS, CK plants underwent abiotic stress, as assessed by H_2_O_2_ and MDA levels; however, SA applications inhibited this effect. Ultraviolet radiation during FIS as shown in Supplementary Table 2 is proof of environmental stress that *M. domestica* plants suffer during FIS at native conditions. The SA treatment alleviate the stress in *M. domestica* plants caused by ultraviolet radiation and positively regulate leaf pigments and inhibit abiotic stress (Mahdavian et al., 2008a,b). The SA-treated leaves maintain cellular redox homeostasis (Durner and Klessig, 1995, 1996; Slaymaker et al., 2002); therefore, SA protects the chlorophyll structure and decreases lipid peroxidation (Uzunova and Popova, 2000), and loss of photosynthetic activity.

The biosynthesis of photosynthetic pigments is linked. The SA-treated group accumulated protoporphyrin by triggering the production of aminolevulinic acid, which is further converted to mg-protoporphyrin by the incorporation of magnesium into the center of the pyrrole ring. Next, the assembly of protochlorophyllide occurs by the reduction of the fourth pyrrole ring, which is then converted to chlorophyllide derivatives by the enzyme protochlorophyllide reductase (Reinbothe and Reinbothe, 1996). Chlorophyll and pheophytin are further synthesized from these derivatives, which initiate electron transfer (Shah et al., 2019a). Salicylic acid also increases the carotenoid content that chains light-absorbing phytochrome machinery and photoprotection. These circumstances enable the leaf to operate photosynthetic functions and initiate ascorbic acid production. This combined effect regulate leaf weight, leaf expansion and early photoperiodic fulfillment, which triggered *MdMED80*, *−81*, *−3*, and *−41* (Figure 10), *MdMED80*, and *MdMED81* to induce flowering in response to light quality (Bäckström et al., 2007). We also hypothesized that, owing to the positive regulation of health-related markers and leaf expansion, the canopy density increases, resulting in a decreased ratio of red to far-red light. The ability of an alteration in light quality to trigger a series of responses is termed shade avoidance syndrome. During this response, the stems lengthen at the expense of leaf expansion, and flowering is triggered (Halliday et al., 1994; Ballaré, 1999). PhyB is a photoreceptor for red/far-red light and plays an important role in response to light protection. The PhyB signal is transmitted to *MdMED80* and *MdMED81*, and it regulates FT expression under appropriate light conditions (Aukerman et al., 1997; Devlin et al., 1998; Cerdán and Chory, 2003). In our results (Figure 10), the *MdMED3* and *MdMED41* homologs of *CRP* were significantly upregulated soon after the SA treatment, which supports the early flowering phenotype (Supplementary Figure 1). *MdMED3* and *MdMED41* are newly identified flowering-regulator genes having regulatory target steps both downstream and upstream of the key flowering regulators as well as the FT florigen (Imura et al., 2012) in leaf tissues (Takada and Goto, 2003; Kobayashi and Weigel, 2007; Adrian et al., 2010; Imaizumi, 2010). The FT florigen protein is expressed in the leaf, goes through the phloem to the SAM, and then stimulates several positive floral regulatory genes to initiate flower formation (Corbesier et al., 2007; Jaeger and Wigge, 2007; Mathieu et al., 2007; Notaguchi et al., 2008). *MdMED3* and *MdMED41* are required for the proper regulation of fruitfull, suppressor of overexpression of constans 1, apetala1, twin sister of FT, FT, and bZIP protein FD as well as the downregulation of flowering locus C (Imura et al., 2012). Three factors contribute to the high levels of FT protein production. First, there is the coordinated expression of FKF1 and GI, which is caused by the circadian clock. Second, there is the stabilization of the FKF1–GI complex by *MdMED80* and *MdMED81*. Finally, there is the stabilization of the CO protein by *MdMED80* and *MDMED81*. This whole process takes place in the leaves. Once FT is produced, it migrates to the buds, where it triggers the developmental processes that lead to flower production. Therefore, the enhanced regulation of leaf health-related markers, as well as increased leaf growth and expansion, allow the capture of sufficient light to stimulate *MdMED80* and *MdMED81* signaling and, in turn, initiate the flowering process. This was in strong agreement with our flowering phenotype observations (Supplementary Figure 1). This implies that maximum leaf pigmentation and expansion result in vigorous and profuse flowering during the FIS.

**FIGURE 11:**
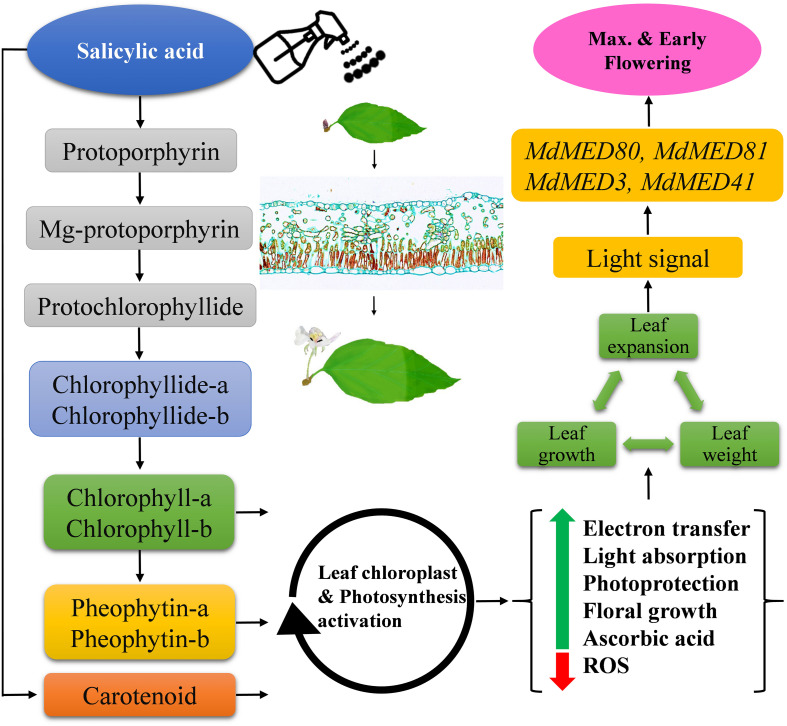
Schematic illustration of the effects of salicylic acid treatments on leaf health-related markers, expansion and early flowering induction in *Malus* × *domestica* during the flower-induction stage. Green and red arrows represent upregulation and downregulation, respectively, while black arrowheads point toward the next biological phase.

In our histological study (Figure 9), maximum leaf thickness, midrib width and area in SA-treated plants during the FIS confirmed the enhanced leaf morphological indices and photosynthetic pigment levels. These may have resulted from SA, xyloglucan endotransglucosylase/hydrolase, a potential target of angustifolia, *MdMED80* and *MdMED81* genes that regulate leaf width through cell-wall loosening and cell expansion (Kim et al., 2002; Rose et al., 2002; Xu and Li, 2011). In summary, SA applications enhanced *M. domestica* leaf micromorphology to produce signals involved in the flowering process, as shown in Figure 11.

Plants are complex systems that require a whole range of processes to satisfy their needs and increase their yield. Some specific functions vary from plant to plant, such as flowering behavior in *M. domestica*, which is related to growth behavior. The flowering behavior of annual plants is different than those of biennial and perennial plants. Similarly, long-day and short-day plants may bear flowers owing to different physiological behaviors. Therefore, plant species do not flower at the same time of year (Jung and Müller, 2009; Xing et al., 2014; Morente-López et al., 2018). Consequently, plants flower in all four seasons, such as *Iris germanica* in summer (Xu et al., 2017), *Chrysanthemum indicum* in fall (He et al., 2016), the *Galanthus nivalis* in winter (Weryszko-Chmielewska and Chwil, 2016) and tulip in spring (Sagdic et al., 2013). The plant’s foliage gathers information from consistent seasonal environmental factors, with day length being the most predictable. The duration of daylight increases in spring and summer before decreasing in fall and winter. This pattern depends on the earth’s motion around the sun, which occurs the same way at the same time every year. Because photoperiod is so predictable, it is the main signal that plant leaves rely on to keep track of the seasons and to flower at the appropriate time. Lorenzo et al. (2019) reported that shade delays flowering, but under normal consistent light conditions, the next most predictable factor that delays flowering is lower leaf health and expansion, because these leaves cannot capture the required light quantity. Thus, SA acts as a potential regulator of leaf health during the FIS and may influence flowering.

## Conclusion

In summary, the current study showed that SA improved leaf health-related marker and leaf growth, which are critical during the FIS in *M. domestica*. We showed that, during the FIS, CK plants exhibited alternate bearing and a late flowering phenotype owing to stunted leaf growth, increase ROS production, deteriorated plant pigments, and weak histological traits, confirming the poor performance of *M. domestica* leaves during the FIS and hence delayed flowering phenotype. Salicylic acid applications during the FIS induced flowering and overcame the alternate bearing and late flowering phenotype. This study helped to explore the roles of SA and importance of leaf assessments during the FIS. We recommend that improvement of leaf profile be used as a primary tool of plant breeders to assess floral induction in plants.

## Data Availability Statement

The original contributions presented in the study are included in the article/Supplementary Material, further inquiries can be directed to the corresponding author/s.

## Author Contributions

LX, MH, NA, YD, and KS designed and conceived the project. KS, LX, LC, PJ, and CZ carried out the experimental work. LX, KS, SK, MM, NA, and XR carried out the data analysis. KS and LX performed the manuscript writing. All authors have read and approved the final version of the manuscript.

## Conflict of Interest

The authors declare that the research was conducted in the absence of any commercial or financial relationships that could be construed as a potential conflict of interest.

## Footnotes

1 https://www.graphpad.com/quickcalcs/molarityform/

## Supplementary Material

Supplementary Figure 1 Flowering comparison of *Malus* × *domestica* on 10th April 2019 in response to CK and SA application. **(A–D)** CK flowering phenotype, **(E–H)** SA flowering phenotype.

Supplementary Figure 2 Phylogenetic tree of Med genes from *Arabidopsis* and *Malus* × *domestica*. The unrooted phylogenetic tree was constructed with IQTREE using the Maximum Likelihood method. The bootstrap test was performed with 1000 iterations.

Supplementary Figure 3 Predicted dimensional structures of selected MdMed proteins in *Malus* × *domestica*.

Supplementary Table 1 Primers used in this study.

Supplementary Table 2 Monthly meteorological statistics of experimental site.
