# Supplementary material for: Regulation of Flowering Time by Improving Leaf Health Markers and Expansion by Salicylic Acid Treatment: A New Approach to Induce Flowering in Malus domestica
Source: Front Plant Sci. 2021 Jul 19;12:655974. doi: 10.3389/fpls.2021.655974 (PMC8328039; doi:10.3389/fpls.2021.655974)
Supplement: Supplementary Figure 1 — Flowering comparison of Malus × domestica on 10th April 2019 in response to CK and SA application. (A–D) CK flowering phenotype, (E–H) SA flowering phenotype. [file Data_Sheet_1.docx]

Supplementary Material


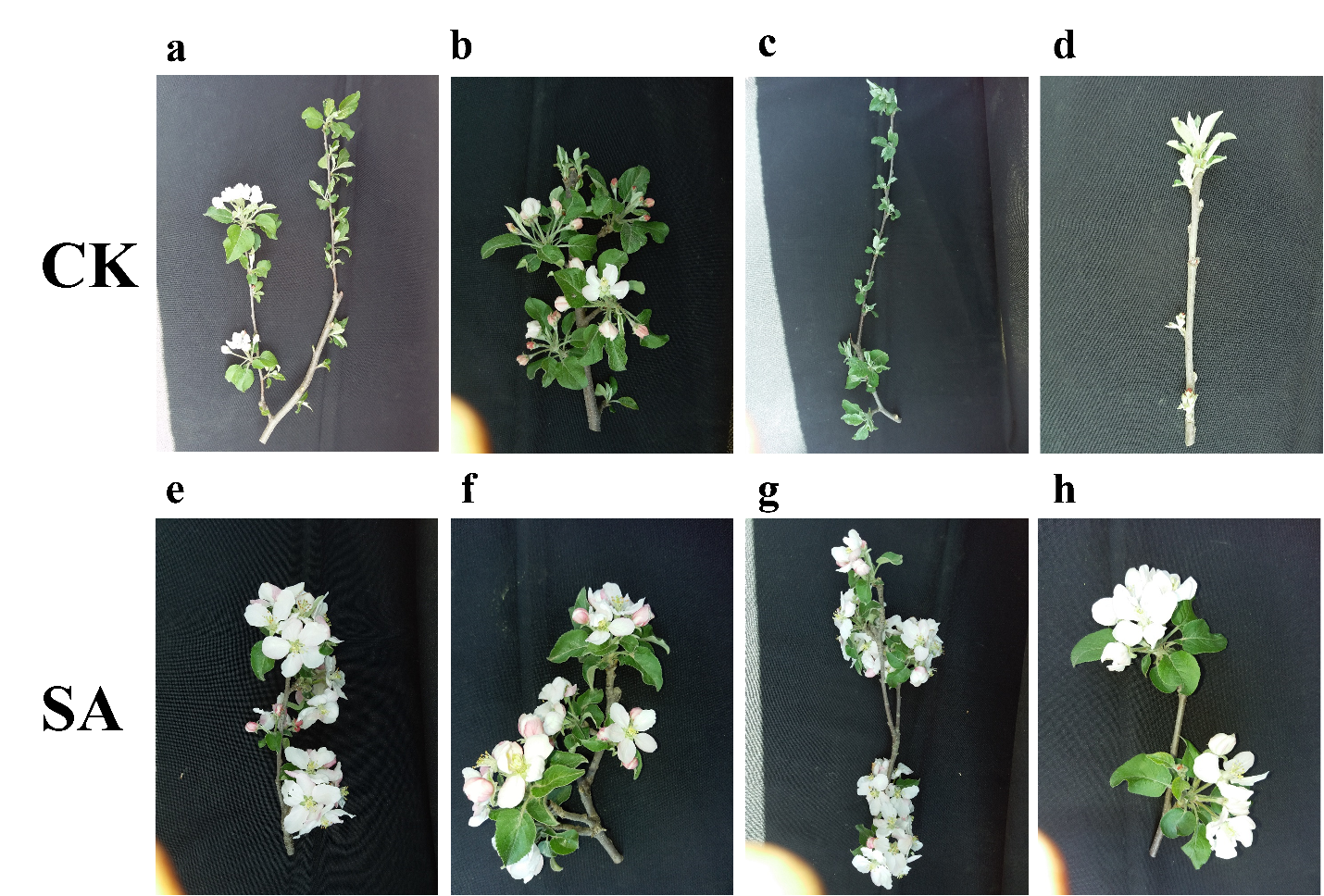


**Figure S1.** Flowering comparison of *Malus × domestica* on 10th April 2019 in response to CK and SA application. (a-d) CK flowering phenotype, (e-h) SA flowering phenotype.


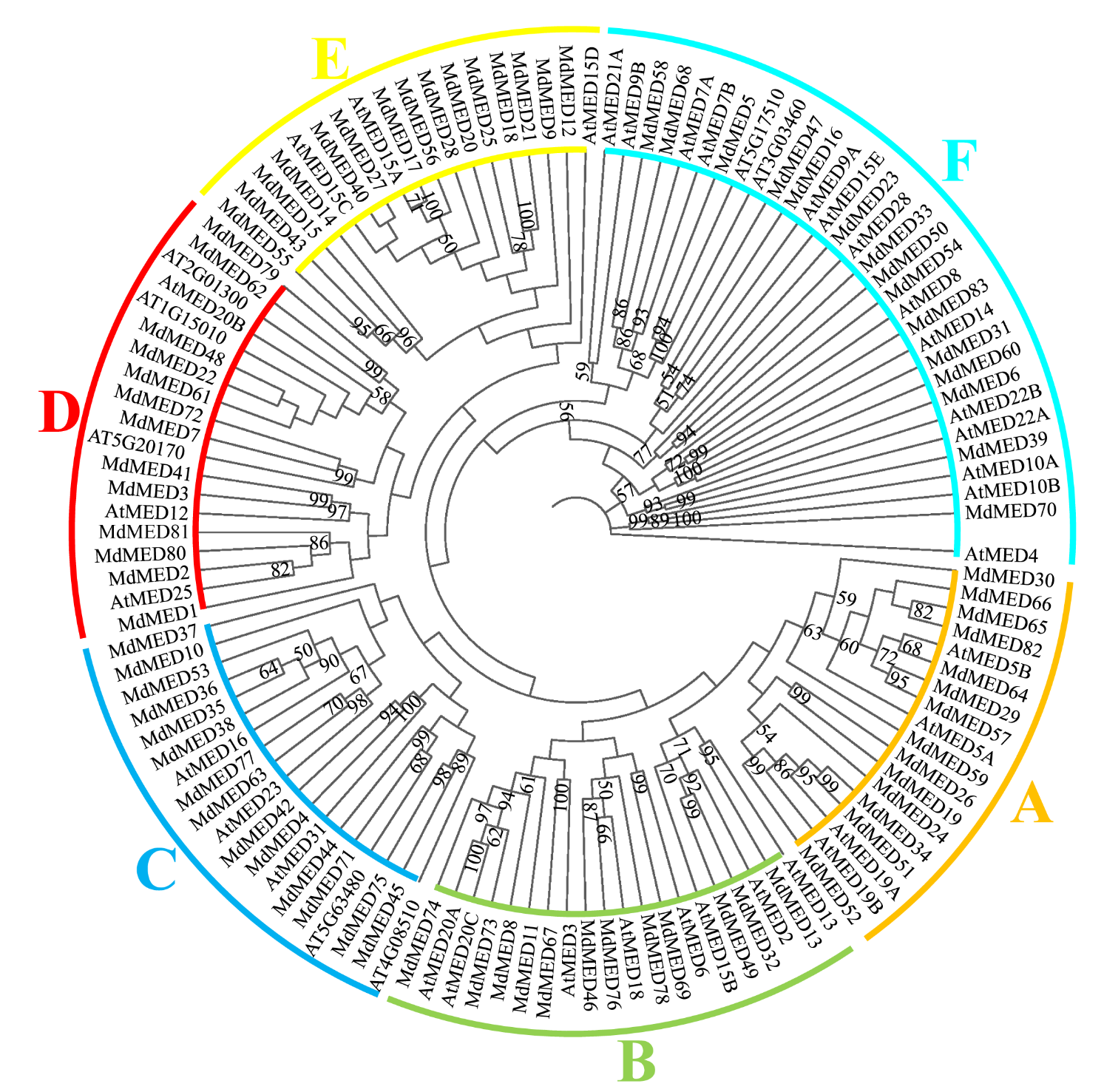


**Figure S2.** Phylogenetic tree of MED genes from *Arabidopsis* and *Malus × domestica*. The unrooted phylogenetic tree was constructed with IQTREE using the Maximum Likelihood method. The bootstrap test was performed with 1000 iterations.

**
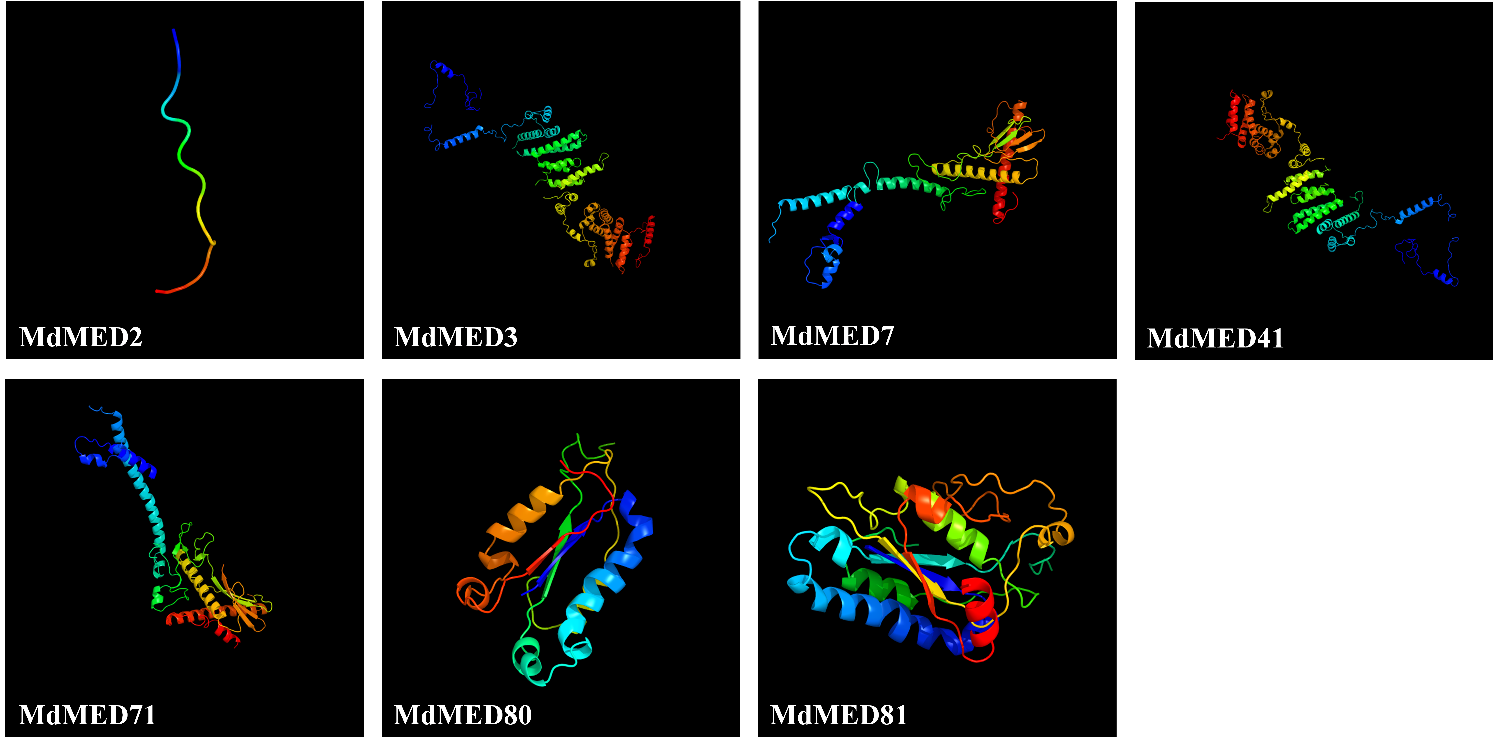
**

**Figure S3.** Predicted dimensional structures of selected MdMED proteins in *Malus × domestica*.

**Table S1.** Primers used in this study.

| **Gene ID** | **Gene Name** | **Primer sequence (5’-3’)** |
| --- | --- | --- |
| MD01G1005800 | *MdMED2* | F : GCATTGGAGGAGAAGGAGAAGAAGA |
|  |  | R : GCTCGGGAACGCTCATCTTGT |
| MD01G1060600 | *MdMED3* | F : TGGACGCTGCTGGAAGATGGT |
|  |  | R : ATCCTTTCGTCTCACCCTCACAGT |
| MD02G1034800 | *MdMED7* | F : TACGACGAGAAGCGGCAGAGTT |
|  |  | R : TGCCATTGCCACTGCGTTGC |
| MD07G1137100 | *MdMED41* | F : ACTGCGTCACTGCCACTAAGGA |
|  |  | R : GCAGAAGCCGCACAACATACCA |
| MD15G1175600 | *MdMED72* | F : GCCAAACCCGAGCATCTGACAT |
|  |  | R : GCCAAGTCGCAATCGTATCTGTTCA |
| MD16G1046800 | *MdMED80* | F : TGCCGCTTGATTGGAATGCTGTT |
|  |  | R : ATGTGTTGTTGCTGACCGCCTAC |
| MD16G1047200 | *MdMED81* | F : GGACCTGTAATGAATCGGCAACCAA |
|  |  | R : GGAAGGAGGACGGACAGATGGAAT |

**Table S2.** Monthly meteorological statistics of experimental site.

| **Qianyang, Baoji, Shaanxi, China** | **Day** | **Night** | **Precipitation** | **Rain days** | **Snow days** | **Sun hours** | **Wind force in bft** | **UV-index** |
| --- | --- | --- | --- | --- | --- | --- | --- | --- |
| **Jan** | 4 ºC | -7ºC | 1 mm | 2 | 2 | 9 | 2 | 2 |
| **Feb** | 7 ºC | -3 ºC | 3 mm | 2 | 2 | 9 | 2 | 3 |
| **Mar** | 13 ºC | 2 ºC | 7 mm | 6 | 1 | 9 | 2 | 3 |
| **Apr** | 19 ºC | 6 ºC | 24 mm | 10 | 0 | 9 | 2 | 4 |
| **May** | 23 ºC | 9 ºC | 26 mm | 12 | 0 | 11 | 2 | 5 |
| **Jun** | 27 ºC | 14 ºC | 26 mm | 12 | 0 | 12 | 2 | 6 |
| **July** | 29 ºC | 17 ºC | 48 mm | 16 | 0 | 11 | 2 | 6 |
| **Aug** | 28 ºC | 15 ºC | 31 mm | 15 | 0 | 9 | 2 | 5 |
| **Sep** | 22 ºC | 11 ºC | 41 mm | 14 | 0 | 8 | 2 | 5 |
| **Oct** | 28 ºC | 7 ºC | 15 mm | 9 | 0 | 9 | 2 | 3 |
| **Nov** | 11 ºC | 1 ºC | 8 mm | 5 | 1 | 7 | 2 | 2 |
| **Dec** | 5 ºC | -5 ºC | 1 mm | 2 | 1 | 7 | 2 | 2 |
